# Two-Year Entomological Survey of Mosquito Fauna in the Attica Region, Greece: Species Composition

**DOI:** 10.3390/insects16040406

**Published:** 2025-04-12

**Authors:** Marina Bisia, Georgios Balatsos, Maria Sakellariou Sofianou, Stavroula Beleri, Nikolaos Tegos, Evangelia Zavitsanou, Vasileios Karras, Dimitra Kollia, Antonios Michaelakis, Eleni Patsoula

**Affiliations:** 1 Laboratory of Insects and Parasites of Medical Importance, Scientific Directorate of Entomology and Agricultural Zoology, Benaki Phytopathological Institute, 14561 Kifissia, Greece; m.bisia@bpi.gr (M.B.); g.balatsos@bpi.gr (G.B.); m.sofianou@bpi.gr (M.S.S.); e.zavitsanou@bpi.gr (E.Z.); v.karras@bpi.gr (V.K.); a.michaelakis@bpi.gr (A.M.); 2 Unit of Medical Entomology, Laboratory for Surveillance of Infectious Diseases, Department of Public Health Policy, School of Public Health, University of West Attica, 11521 Athens, Greece; smpeleri@uniwa.gr (S.B.); ntegos@uniwa.gr (N.T.)

**Keywords:** *Aedes cretinus*, *Aedes geniculatus*, *Anopheles superpictus*, *Culex perexiguus*, *Culicidae*, *Culex perexiguus*, mosquitoes

## Abstract

Vector-borne diseases significantly impact global public health, with mosquitoes playing a critical role in the transmission of various pathogens. This study focused on the mosquito fauna in the Attica region of Greece, conducting a two-year entomological survey from March 2021 to December 2022 as part of an ongoing mosquito-management program. The research employed stratified random sampling to establish 57 adult traps across the region, with additional traps on the islands of Argosaronikos and Kythira island. The BG-sentinel traps, enhanced with CO_2_ to attract multiple mosquito species, were utilized for mosquito collection. Morphological identification of the collected mosquitoes revealed the presence of various species, with *Aedes albopictus*, *Culex pipiens* s.l., and *Culiseta longiareolata* being the most prevalent. Notably, all of our traps tested 100% positive for these species. Molecular techniques, including PCR amplification of ITS2 and COI genes, confirmed species identification. The findings highlight significant variations in species composition across different locations and emphasize the presence of invasive species such as *Aedes albopictus*, posing public health concerns. This study underscores the importance of continuous mosquito surveillance and integrated management strategies to mitigate the risk of mosquito-borne diseases in the Attica region. The results contribute to the development of evidence-based mosquito-control programs, which are essential for safeguarding public health in urban and peri-urban environments.

## Simple Summary

This study, as part of an integrated mosquito-management program, investigated mosquito species distribution in the Attica region of Greece, an important factor in controlling vector-borne diseases like West Nile virus. Over two years, traps were set up across various locations to catch mosquitoes. It was found that the Asian tiger mosquito (*Aedes albopictus*) and the common house mosquito (*Culex pipiens* s.l.) are widespread in the region. Some mosquito species were identified using DNA analysis to ensure accuracy. The study detected differences in mosquito species distribution across different locations over the season, emphasizing the need for ongoing monitoring and control efforts to protect public health. This research helps to better plan integrated mosquito-control programs to reduce the risk of disease transmission in the region.

## 1. Introduction

Vector-borne diseases pose a significant challenge to public health due to their widespread distribution and substantial impact on both human and animal populations worldwide [1]. These diseases, transmitted by various vectors, influence quality of life, contributing to morbidity and mortality on a broad scale [2,3]. Among the plethora of vectors, mosquitoes emerge as key players in this health burden, serving as carriers for pathogens responsible for diseases such as malaria, West Nile virus, Dengue, Chikungunya, and Zika virus [4].

Mosquito control should be based on integrated pest management, relying on the combined use of several mosquito-control tools selected according to evidence provided by surveillance [5]. The establishment of a consistent, reliable, and sustainable long-term surveillance system is, therefore, a critical component to any successful mosquito-control program in order to make informed decisions and respond appropriately to changing mosquito populations. Given the threat posed by vector-borne diseases, entomological studies play a crucial role in this process, serving as early warning systems for the presence of mosquitoes and associated diseases while also supporting the implementation of integrated mosquito-management strategies [6,7,8,9]. Understanding the distribution, abundance, and species composition of mosquitoes becomes increasingly significant when considering the heightened risk of vector-borne diseases associated with the expansion of native mosquito species and the invasion of alien species [10,11]. This risk is further amplified by factors such as climate change, the globalization of travel and trade, changes in land use, and urbanization [12]. These processes necessitate a comprehensive investigation into the factors driving the increase of mosquito vectors and their implications for public health, reinforcing the necessity for proactive measures to mitigate the impact of mosquito-borne diseases [13,14].

Globally, there are thousands of documented mosquito species, with a subset acting as proven vectors of pathogens [13,15,16,17,18]. In Greece, the documentation of mosquito fauna has been undertaken by dedicated researchers. The first invasion of *Aedes albopictus* in Greece occurred at Corfu and Thesprotia in 2003 [19], signaling a pivotal moment in the country’s vector ecology. Subsequently, this event was followed by its establishment in the Attica region 5 years later, highlighting the need for a focused examination of mosquito populations in the largest region of Greece, which accommodates half of Greece’s population [20].

The Attica region, with its unique combination of urban and rural landscapes, serves as a microcosm of the challenges posed by mosquito-borne diseases [21,22]. Understanding and monitoring the mosquito fauna in this densely populated region is of great importance, given its potential impact on public health. This understanding is not only vital for the residents of Attica but also holds broader implications for managing the overall health and well-being of Greece’s population.

In every municipality across Greece, including Attica, mosquito-control programs are overseen by local authorities. Routine biocide applications are conducted by private companies contracted by each municipality or region. Expert scientific personnel within local authorities use surveillance data to guide the timing and type of additional interventions and to evaluate the effectiveness of these biocide applications. As part of this program, our team conducted a comprehensive surveillance initiative across the Attica region, using an intensive trapping system to monitor mosquito species and identify new or uncommon species. The aim of this study was to update and expand the existing data on mosquito fauna composition within the Attica region for the years 2021 and 2022.

## 2. Materials and Methods

### 2.1. Methodological Approach for Mosquito Trap Site Selection

The geostatistical method of stratified random sampling was employed to choose representative sites for mosquito traps across the Attica region. This method was applied to various land uses throughout the region, guided by entomological requirements. The selection process utilized Corine Land Cover (CLC 2018) data, initially identifying 50 representative sites (trap locations) (Supplementary Figure S1; created with ArcGIS Pro (3.0.0), available from Esri (Athens, Greece) (Marathon Data, GR)).

The Attica region is administratively divided into eight regional units (RU). So, the number of traps selected for each RU was in accordance with the unit’s land coverage, and the sampling effort was equivalent for all the RU. Thus, to refine the selection of trap locations, we analyzed entomological data from previous years [22,23]. Additionally, input was asked from all 58 municipalities to suggest suitable sites. In nearly all situations, the initially chosen locations were deemed appropriate, ensuring a precise geostatistical representation. In cases where setting traps at the original locations was unfeasible (e.g., military camps, no electrical supply, etc.), nearby alternatives were selected to ensure accurate entomological monitoring.

In total, 57 adult traps were established across the region, facilitating comprehensive surveillance of mosquito populations. In addition, eight traps were established in the islands of Argosaronikos and Kythira, which administratively belong to the Attica region. The trap type selected was the BG-sentinel trap, equipped with the BG-lure. The specific trap was selected because it is highly effective at attracting *Aedes* mosquitoes. To further enhance its efficiency and ensure the collection of mosquitoes from other genera, such as *Culex* and *Anopheles*, a constant flow of CO_2_ was incorporated. This trapping system has been successfully tested and validated in previous studies, demonstrating its reliability for capturing a diverse range of mosquito species [7,22].

### 2.2. Collection of Samples

The current study was carried out within the research project for the entomological surveillance of mosquitoes in Attica region from March 2021 to December 2022. Each week, a staff member had the responsibility to collect the samples while recording every detail about the trap’s function. After collection, the samples from the BG traps were stored on ice for their transportation to the laboratory of Insects and Parasites of Medical Importance at the Benaki Phytopathological Institute.

### 2.3. Morphological Identification

After storing for about 2 h in freezing temperatures (approximately −20 °C) to ensure the insects’ death, each sample was laid on a petri dish and observed, with the help of entomological forceps, under a stereoscope (NikonSMZ745, Nikon, Tokyo, Japan). Firstly, the presence of mosquitoes in the sample was ensured since BG traps can also attract other insect taxa. If mosquitoes were present in the sample, the individuals’ sex was identified, and then, adult mosquitoes were morphologically classified at species level based on specific characteristics using identification keys [24]. If the sample was partly damaged and if crucial morphological characteristics necessary for identification, such as wings and legs, were missing, identification could only be determined up to the genus level.

### 2.4. DNA Extraction and Polymerase Chain Reaction (PCR) Amplification

A total of 11 adult mosquitoes that were morphologically identified up to genus level were further examined at the molecular level to verify species identification. Additionally, the sibling species *Anopheles maculipennis* s.s. and *Anopheles sacharovi* were verified through barcoding. DNA was extracted from individual whole adults using the NucleoSpin Tissue, DNA Mini kit (MACHEREY-NAGEL, Düren, Germany), following manufacturers’ instructions. The nuclear ribosomal spacer gene ITS2 was amplified by PCR using two different protocols: one targeting the internal transcribed spacer 2 (ITS2) region from the nuclear ribosomal DNA using 5, 8S, and 28S primers and a second one amplifying part of the mitochondrial cytochrome oxidase I gene (COI) using primers C1-J-1718 and C1-N-2191, with the related PCR protocols being carried out as previously described [25].

Products were electrophoresed and sent for sequencing analysis (CEMIA, SA, Larissa, Greece). Similarity with sequences available in GenBank was assessed using the Basic Local Alignment Tool (BLAST) Blastn, and sequences were aligned using the CLUSTAL omega software (1.2.4) multiple sequence alignment tool (EMBL-EBI).

## 3. Results

The morphological identification of mosquitoes collected from adult traps unveiled the prevalence of two predominant species: the Asian tiger mosquito and the common mosquito (*Culex pipiens* s.l.). Both mosquito species established populations throughout the Attica region for 2021 and 2022. Additionally, *Culiseta longiareolata* was captured in all the traps across various locations, indicating its widespread presence. In addition, the following species were collected in various locations across the Attica region, throughout the two-year survey: *Ae. caspius*, *Ae. cretinus*, *Ae. detritus*, *Ae. dorsalis*, *Ae. geniculatus*, *Ae. vexans*, *Ae. pulcritarsis*, *Ae. zammitti/mariae*, *Anopheles algeriensis*, *An. claviger*, *An. maculipennis s.s*, *An. sacharovi*, *An. superpictus*, *Coquillettidia richiardii*, *Cs. annulata*, *Cs. morsitans*, *Cx. mimeticus*, *Cx. perexiguus*, *Cx. theileri*, and *Uranotaenia unguiculata*. In Table 1, it is evident that the majority of the captured mosquito species are limited to a few locations across the region.

**Figure 1:**
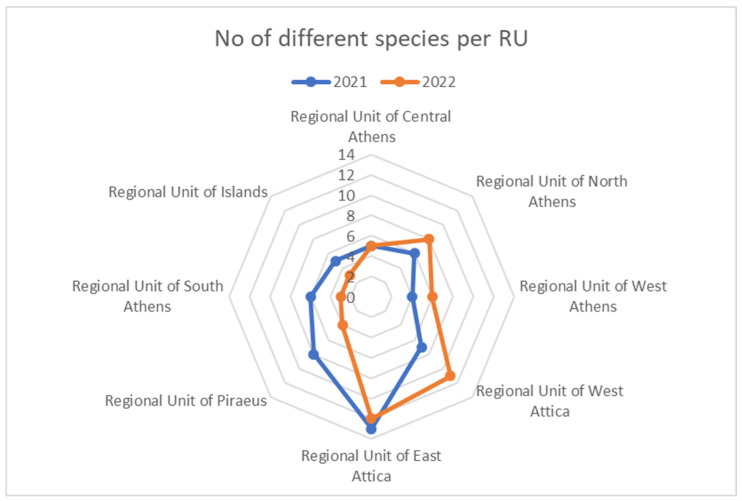
The distribution of different mosquito species collected during the years 2021–2022 at the different RUs of Attica region.

Taking into account that each RU covers a different land area and has a suitable number of traps positioned across its territory, we can point out that East Attica RU had the highest mosquito species diversity in both 2021 and 2022 (Supplementary Table S1, Figure 1).

In contrast, the West Athens RU recorded the lowest species diversity in 2021, while the South Athens RU and the Islands RU had the lowest diversity in mosquito species in 2022. We also observed that some traps collected a great number of different mosquito species, like trap 46 in Sxoinias-Marathonas, which collected 12 different mosquito species in 2021 and 2022. The majority of the traps collected only the main three mosquitoes—*C. pipiens* s.l., *Ae. albopictus*, and *Cs. longiareolata* (40 traps for 2021 and 48 for 2022 out of 65 total BG traps across the Attica region).

Molecular techniques were utilized for multiple mosquito samples where identifiable characteristics were missing, rendering their morphological identification to species level impossible. In 2021, two samples were morphologically identified as *Culex* spp. and molecularly typed as *Cx. theileri*. Additionally, one sample was identified as *Anopheles* spp. and one as *Aedes* spp., with molecular analysis confirming them as *An. algeriensis* and *Ae. zammitii* or *Ae. mariae*, respectively. For the next year, 2022, three samples were morphologically identified as *Aedes* spp. and subsequently molecularly confirmed as *Ae. pulcritarsis*, *Ae. Detritus*, and *Ae. albopictus*. Additionally, one sample was identified as *Anopheles* spp. and one as *Culiseta* spp., with molecular analysis confirming them as *An. sacharovi* and *Cs. morsitans*, respectively. In both years, collected *Anopheles maculipennis* s.s. and *Anopheles sacharovi* adults were also molecularly verified due to their similar morphological characteristics. This combined morphological and molecular approach allowed for the accurate identification of mosquito species that otherwise would have been classified only at genus level.

## 4. Discussion 

The findings of our entomological survey in the Attica region underscore the significant presence and distribution of mosquito species, particularly *Ae. albopictus* and *Cx. pipiens* s.l. These results align with previous studies indicating the prevalence of these species in urban and peri-urban environments [23]. The robust population of *Ae. albopictus* highlights the establishment and persistence of this invasive species in the region [20] This invasive mosquito species, known for its adaptability to diverse environments and its role as a vector for several arboviruses, poses a considerable public health concern [26].

The coexistence of *Ae. albopictus* and native *Cx. pipiens* s.l. in the Attica region highlights the complexity of mosquito communities in urban areas [27]. While *Ae. albopictus* breeds in containers and artificial water-holding habitats commonly found in urban landscapes [28], *Cx. pipiens* s.l., known as the common house mosquito, exhibits a broader habitat range, including natural and artificial breeding sites [29]. The observed variations in species across different trapping locations suggest localized environmental factors influencing mosquito species composition, which aligns with previous research in the area [22,30]. In Europe, including Greece, *Cx. pipiens* and *Cx. modestus* are the primary vectors of WNV [31]. Recently, *Cx. perexiguus* has also been evaluated as a potential bridge vector due to its microbiota and ecological behavior [32].

Our survey identified *Anopheles* mosquitoes in specific, expected locations across the Attica region, consistent with previous studies and within their common habitats [22]. Importantly, we did not observe *Anopheles* populations outside of these established environments, reaffirming their known distribution patterns in the area. As the primary vectors of malaria—one of the most impactful infectious diseases—monitoring *Anopheles* populations remains crucial [33]. Especially, the identified species (*An. claviger*, *An. maculipennis* s.s., *An. sacharovi*, and *An. superpictus*) are the main vectors of human malaria in Europe and could also be vectors of different pathogens like *An. maculipennis* s.s., which is a secondary vector of *Dirofilaria* spp. [7].

A comparison of our results with studies conducted over the past decade revealed that while all previous investigations identified the prevalence of common mosquito species and the Asian tiger mosquito, our study highlighted notable variations in species composition across different locations within the Attica region during 2021 and 2022 [22,34,35]. Our findings also indicate a widespread distribution of *Ae. caspius* across numerous sites—a pattern not observed in prior studies within the region [35]. This species, along with *Ae. vexans* and *Cx. perexiguus*, both detected in our research, are known vectors of Rift Valley fever virus outside Europe [7]. Additionally, *Cx. perexiguus* has been documented as both a naturally infected and competent vector of Sindbis virus, while *Cs. morsitans* is a known vector of Sindbis virus and a secondary vector of *Dirofilaria* spp. Our results that identified additional mosquito species that re-emerged in the area after a long period of absence of recording expand on the current understanding provided by previous projects in the area [22,34,35]. This study documented a broader range of mosquito species and mapped their locational presence—an essential step, as various mosquito species are confirmed or implicated as vectors of multiple pathogens.

Our molecular analyses provided valuable insights into mosquito species identification, particularly when morphological characteristics were insufficient for precise species identification [25,36]. The application of PCR amplification targeting the ITS2 and COI genes enabled accurate species identification, overcoming any morphological limitations. This approach validated the morphological identification of specimens and facilitated the detection of even new invasive species [37].

The consistent surveillance of mosquito populations through entomological studies serves as a crucial component of integrated mosquito-management strategies [38]. By monitoring mosquito abundance and species composition, public health authorities can implement timely interventions to mitigate the risk of mosquito-borne diseases. Strategies such as larval habitat reduction, insecticide application, and community engagement initiatives can effectively target mosquito populations and reduce the incidence of vector-borne diseases in endemic areas [39].

Furthermore, our study highlights the importance of ongoing vigilance and surveillance efforts in regions like Attica, where the convergence of urbanization, climate change, and globalization may exacerbate the risk of mosquito-borne diseases [40,41]. As urban areas expand and human activities continue to alter landscapes, mosquito populations are likely to shift in their geographic range and seasonality, necessitating adaptive and proactive approaches to disease prevention and control [42,43]. Especially, the recent example of the newly established *Ae. aegypti* population in Cyprus [44] increases concern of a similar event occurring in Greece. The continuous monitoring of the mosquito species in the area can be an early warning system for that invasion.

## 5. Conclusions

In conclusion, our ongoing research contributes to the body of knowledge on mosquito ecology and public health in the Attica region. One limitation of this research is the use of a single type of adult mosquito trap, which may have restricted the diversity of the species captured compared to other types of adult traps. Additionally, the study’s focus did not extend to collecting eggs or larvae, so some species may have gone undetected. Establishing a consistent, reliable, and sustainable long-term surveillance system is thus an imperative element of any effective mosquito-control program. By combining traditional entomological methods with molecular techniques, we provide a comprehensive assessment of mosquito species composition and distribution. The insights gained from this study can inform evidence-based decision making and guide the implementation of targeted interventions to reduce the burden of mosquito-borne diseases in urban environments. Continued research and surveillance efforts are essential for addressing emerging threats and safeguarding the health and well-being of local communities.

## Figures and Tables

**Table 1:** Summary of Mosquito Species Collected in the Attica Region and Percentage of Positive Traps for Each Species during the 2021 and 2022 collection periods. The trap positivity rate is defined as the percentage of traps that collected at least one specimen of the target species.

Mosquito Species	Trap Positivity Rate (%) Across	
	2021	2022
*Ae. albopictus*	100	100
*A* *e. caspius*	22	12
*Ae. cretinus*	2	2
*Ae. detritus*	5	8
*Ae. dorsalis*	2	0
*Ae. geniculatus*	2	5
*Ae. vexans*	3	5
*Ae. pulcritarsis*	0	2
*Ae. zammitti/mariae*	3	0
*An. algeriensis*	5	3
*An. claviger*	3	0
*An. maculipennis* s.s.	0	2
*An. sacharovi*	2	3
*An. superpictus*	2	0
*Co. richiardii*	8	3
*Cs. annulata*	11	6
*Cs. longiareolata*	100	100
*Cs. morsitans*	0	2
*Cx. pipiens*	100	100
*Cx. mimeticus*	2	0
*Cx. perexiguus*	2	0
*Cx. theileri*	3	0
*Ur. unguiculata*	0	5

## Data Availability

The original contributions presented in this study are included in the article/Supplementary Material. Further inquiries can be directed to the corresponding author.

## Supplementary Materials

The following supporting information can be downloaded at https://www.mdpi.com/article/10.3390/insects16040406/s1. Figure S1: Map of the Attica region showing land uses types and adult traps locations (yellow dots); Table S1: The traps (with coordinates) where each mosquito species was collected.
