# Supplementary material for: Two-Year Entomological Survey of Mosquito Fauna in the Attica Region, Greece: Species Composition
Source: Insects. 2025 Apr 12;16(4):406. doi: 10.3390/insects16040406 (PMC12027945; doi:10.3390/insects16040406)
Supplement: Supplementary file 1 [file insects-16-00406-s001.zip › insects-3528631-supplementary.pdf]

Supplementary Materials

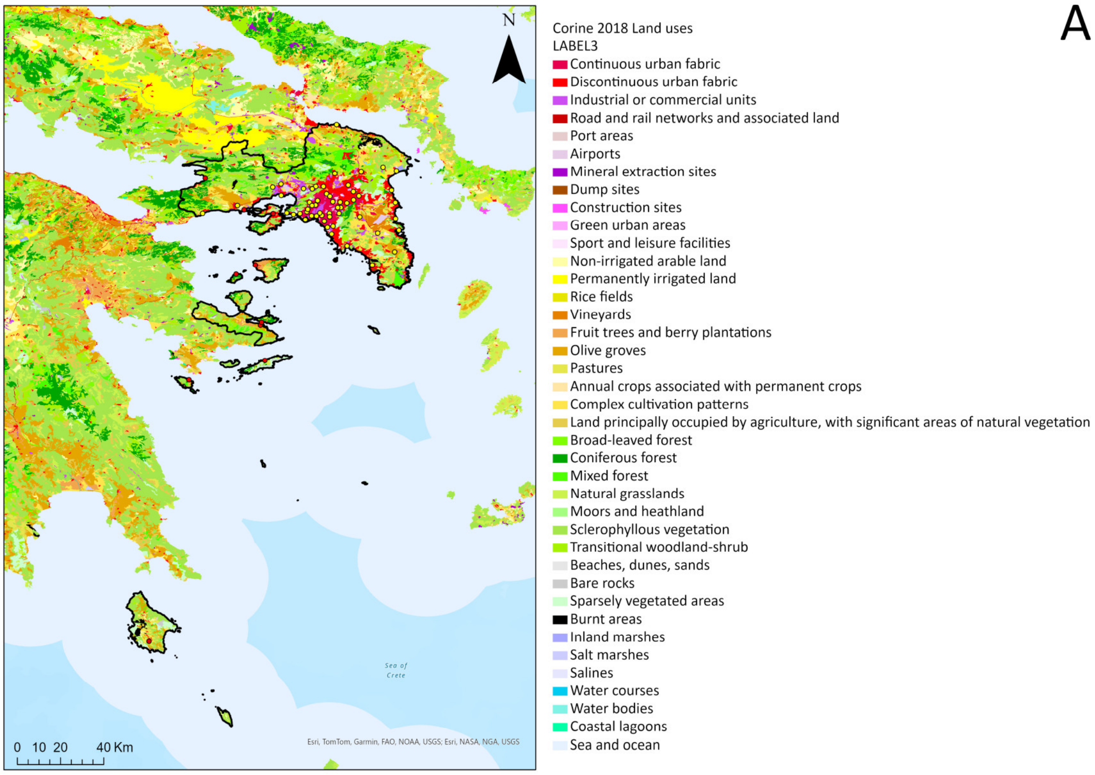

Figure S1. Map of the Attica region showing land uses types and adult traps locations (yellow dots).

Table S1. The traps (with coordinates) where each mosquito species was collected.

Mosquito Species	BG Code	Coordinates		Year	
		X	Y	2021	2022
Ae. caspius	13	24.010484°	37.941965°	x	x
	15	23.710623°	38.075910°	x	
	38	24.007095°	37.802581°	x	x
	46	24.014762°	38.142285°	x	x
	49	23.764983°	38.335280°	x	x
	54	23.612862°	37.964025°	x	x
	55	23.558504°	37.966032°	x	
	56	23.661340°	37.954354°	x	
	6	23.723259°	38.045165°	x	x
	66	23.2187440°	38.110299°		x
	67	23.381053°	37.979806°	x	x
	69	23.6351010°	37.938013°	x	
	9	23.534732°	38.039269°	x	
	IP3	22.984650°	36.179502°	x	
Ae. cretinus	43	23.834911°	38.054748°	x	x
Ae. detritus	14	23.836315°	38.020168°	x	
	1	23.7832958°	37.9885128°		x
	45	23.993837°	38.060067°		x
	46	24.014762°	38.142285°	x	x
	49	23.764983°	38.335280°		x
	6	23.723259°	38.045165°		x
	64	23.681782°	37.953146°	x	
Ae. dorsalis	55	23.558504°	37.966032°	x	
Ae. geniculatus	9	23.534732°	38.039269°		x
	43	23.834911°	38.054748°		x
	46	24.014762°	38.142285°	x	x
	49	23.764983°	38.335280°		x
Ae. vexans	1	23.7832958°	37.9885128°	x	x
	42	23.6644720°	38,0655434°		x
	43	23.834911°	38.054748°	x	
	52	23.813682°	38.081233°		x
Ae. pulcritarsis	52	23.813682°	38.081233°		x
Ae. zammitti/mariae	65	23.696108°	37.966367°	x	
	55	23.558504°	37.966032°	x	
An. algeriensis	28	24.024745°	37.892658°	x	x
	46	24.014762°	38.142285°	x	x
	49	23.764983°	38.335280°	x	
An. claviger	46	24.014762°	38.142285°	x	
	IP3	22.984650°	36.179502°	x	
An. maculipennis	31	23.534214°	38.085891°		x
An. sacharovi	42	23.6644720°	38.0655434°		x
	46	24.014762°	38.142285°	x	x
An. superpictus	9	23.534732°	38.039269°	x	
Co. richiardii	15	23.710623°	38.075910°	x	
	46	24.014762°	38.142285°	x	x

	5	23.736389°	37.950833°	x	
	61	23.703741°	37.955738°	x	
	63	23.662946°	37.997268°	x	
	66	23.2187440°	38.110299°		x
Cs. annulata	30	23.677694°	37.998686°		x
	33	23.628335°	38.066885°	x	
	35	23.678889°	38.076389°		x
	42	23.6644720°	38.0655434°	x	
	46	24.014762°	38.142285°	x	x
	54	23.612862°	37.964025°	x	
	55	23.558504°	37.966032°	x	
	56	23.661340°	37.954354°	x	
	66	23.2187440°	38.110299°		x
	67	23.381053°	37.979806°	x	
Cs. morsitans	46	24.014762°	38.142285°		x
Cx. mimeticus	4	23.645183°	37.980947°	x	
Cx. perexiguus	38	24.007095°	37.802581°	x	
Cx. theileri	38	24.007095°	37.802581°	x	
	46	24.014762°	38.142285°	x	
Ur. unguiculata	42	23.6644720°	38.0655434°		x
	43	23.834911°	38.054748°		x
	46	24.014762°	38.142285°		x
